# Gastrocnemius Medialis Architectural Properties at Rest and During Stretching in Female Athletes with Different Flexibility Training Background

**DOI:** 10.3390/sports7020039

**Published:** 2019-02-13

**Authors:** Olyvia Donti, Ioli Panidis, Gerasimos Terzis, Gregory C. Bogdanis

**Affiliations:** Sports Performance Laboratory, School of Physical Education & Sport Science, National and Kapodistrian University of Athens, Athens 17237, Greece; ipanidi@phed.uoa.gr (I.P.); gterzis@phed.uoa.gr (G.T.)

**Keywords:** muscle architecture, fascicle length, stretching, skeletal muscle ultrasonoraphy

## Abstract

Background: This study examined gastrocnemius medialis (GM) architectural properties and ankle joint range of motion (ROM) between female athletes with different flexibility training background. Methods: Elite rhythmic gymnasts (*n* = 10) were compared to national level volleyball athletes (*n* = 10). Fascicle length, pennation angle and muscle thickness at the medial and the distal part of GM, and ankle ROM were measured at rest and during 1 min of static stretching. Results: At rest, rhythmic gymnasts displayed longer fascicles compared to volleyball athletes, at the medial (5.93 ± 0.27 vs. 4.74 ± 0.33 mm, respectively, *p* = 0.001) and the distal part of GM (5.63 ± 0.52 vs. 4.57 ± 0.51 mm, respectively, *p* = 0.001), smaller pennation angle at the medial part (22.4 ± 2.5 vs. 25.8 ± 2.4°; respectively, *p* = 0.001) and greater ankle angle (121.7 ± 4.1 vs. 113.2 ± 3.7°, respectively, *p* = 0.001). During the 1 min of static stretching, gymnasts displayed greater fascicle elongation at the distal part (*p* = 0.026), greater maximal ankle dorsiflexion (*p* < 0.001) and muscle tendon junction displacement (*p* < 0.001) with no difference between groups in pennation angles (*p* > 0.145), muscle thickness (*p* > 0.105), and fascicle elongation at mid-belly (*p* = 0.063). Conclusions: Longer muscle fascicles at rest and greater fascicle elongation at the distal part of GM may contribute to the greater ankle ROM observed in rhythmic gymnasts.

## 1. Introduction

Flexibility is considered an important fitness component in several sports and especially those requiring an athlete to move powerfully through a wide range of motion (ROM) [1,2]. Static stretching is commonly used to increase ROM [3], enhance athletic performance [4], and reduce the risk of muscle strain related injury [5]. Transient increases in ROM after static stretching are mainly due to increased stretch tolerance [6], and changes in passive stiffness of the muscle and tendon unit [7]. Chronic static stretching interventions in animals may induce an increase in muscle size and fascicle length through the addition of sarcomeres in series, and in particular at the muscle tendon junction (MTJ) [8,9]. However, long-term joint ROM increases following static stretching in humans are not adequately documented. Simpson et al. [10] found that 6 weeks of overloaded static stretching (stretching in a leg press machine loaded to 20% of the subjects’ maximal voluntary contraction) increased ankle ROM (77 ± 5.4° to 65.5 ± 5.8°, *p* < 0.001) and medial and lateral gastrocnemii fascicle length and thickness. In contrast, Blazevich et al. [11] did not detect any changes in plantar flexors fascicle length, and tendon elongation, following 3 weeks of static stretching training, although stretch training increased by 19.9 ± 10.8% maximum dorsiflexion ROM (*p* = 0.001). Collectively, long-term changes in muscle architectural characteristics and joint ROM, following static stretching remain under question. One reason might be that a parallel change in ROM and muscle architectural characteristics may be a long-term training adaptation. Although genetic predisposition and sport-specific selection may affect muscle architecture and joint ROM [12] examining populations with a chronic flexibility training background may provide additional information on muscle structural adaptations. Along this line, a long-term stretching intervention (15 weeks) in flexibility trained individuals (female gymnasts) resulted in significant hip joint ROM increases after the first 6 weeks of training [13]. 

However, evidence is limited on the effects of long-term flexibility training on muscle architectural characteristics. Thus, the purpose of this study was to examine possible differences in GM architectural characteristics at rest and during 1 min of static stretching, between athletes who have undergone long-term intensive flexibility training and athletes who typically perform significantly less stretching in their everyday training. To this end, we compared female athletes with at least 10 years training background from two different sports: rhythmic gymnastics and volleyball. Elite rhythmic gymnasts typically perform extensive flexibility training [14], while volleyball training does not include the amount of stretching training that is performed in rhythmic gymnastics [15]. The medial gastrocnemius muscle was used, since ankle plantar flexors are both important power generators during jumping [16] and their extensibility is closely linked with ankle joint ROM. Muscle architectural parameters were determined not only at mid-belly, but also at the distal part of the muscle, since the mid-belly might not accurately reflect muscle architecture across the entire gastrocnemius muscle [17].

## 2. Materials and Methods

### 2.1. Participants

Ten elite level, rhythmic gymnasts, were compared to 10 female, national level volleyball players. While both sports involve jumping and landing activities, rhythmic gymnastics training includes extensive daily flexibility training (≈60 min), while volleyball (and most team sports) training includes <10 min of stretching exercises per session [14,18,19]. The groups had similar age, competitive experience, leg length, and calf length (Table 1). Participants had no injuries of the lower limbs including incidences of muscle strains for the past 6 months. The Institutional Ethics Committee approved the study (registration number: 1040, 14/02/2018) in accordance with the Declaration of Helsinki and participants signed an informed consent prior to the start of the study.

### 2.2. Experimental Design

**Figure 1:**
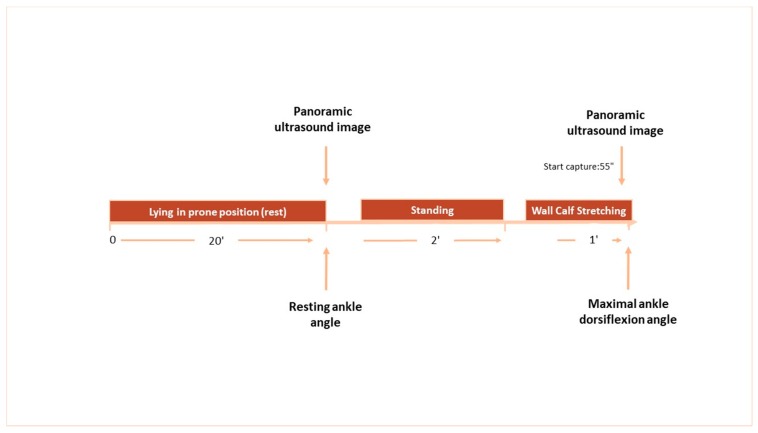
Schematic representation of the study protocol. Measurements of panoramic ultrasound images and ankle joint angles are indicated by arrows.

To compare architectural characteristics of gastrocnemius medialis (GM) and ankle joint ROM in female elite athletes with different stretching background, volleyball and rhythmic gymnastics athletes were tested during the competitive season (April to May) over two sessions. One week prior to the main testing session, one familiarization session took place to get participants familiarized with the testing procedures and obtain anthropometric characteristics. The main testing session included measurements of resting fascicle length, pennation angle, muscular thickness, and ankle joint ROM with the participants lying in prone position. Following 2 min of standing, the same variables were assessed during the 1 min standing calf stretching (Figure 1). 

### 2.3. Anthropometry

Height, was measured to the nearest 0.1 cm with the use of a stadiometer (Seca 208, Hamburg, Germany) and body mass was measured to the nearest 0.1 kg with a calibrated digital scale (Seca 710, Hamburg, Germany). Leg length was measured as the distance between the most prominent point of the trochanter major to the floor with the athletes standing upright. Calf length was measured from the tibiofemoral cleft to the most prominent spot of the medial malleolus.

### 2.4. Resting Muscle Architecture and Ankle Joint Angle

**Figure 2:**
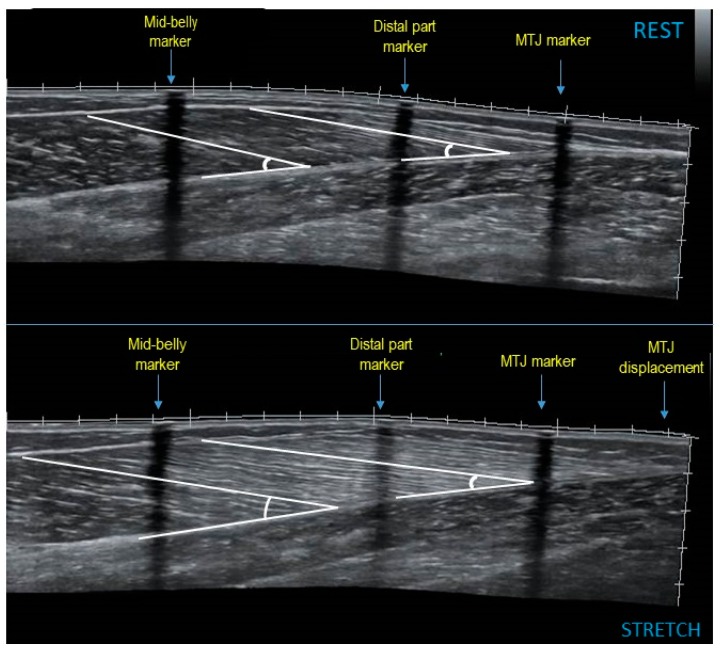
Panoramic sonographic image of a rhythmic gymnast gastrocnemius medialis at rest and during stretching, showing fascicle length and pennation angle at mid-belly and at the distal part of the muscle. MTJ: muscle-tendon junction.

All ultrasound measurements were performed between 9:00 and 12:00, after participants remained in a prone position for 20 min, with the foot hanging freely off the examination bed, to allow fluid redistribution [20]. This ankle position was considered as resting prone position. Architectural characteristics (fascicle length, pennation angle and muscle thickness) of GM of the right leg, were measured at the middle and the distal part of the GM muscle belly: 30% and 50% of the distance from the popliteal crease to the centre of the medial malleolus respectively, marked on the skin through placement of an echo-absorptive marker [21]. The location of the GM musculotendinous junction (MTJ) was identified by ultrasonography and marked on the skin surface through placement of an echo-absorptive marker. Panoramic B-mode ultrasound images from GM were obtained with a 38-mm linear probe using the “i-scape” software of the ultrasound device (10.0-MHz, Product model Z5, Shenzen, Mindray Bio-Medical Electronics Co., Ltd., Shenzen, China). Aquasonic clear ultrasound transmission gel (Parker laboratories, Inc., New Fairfield, New Jersey, USA) provided acoustic contact between the probe and the skin. The transducer was placed 38 mm before the skin marker identifying the 30% of the distance between the popliteal crease and the center of the medial malleolus. The transducer was placed longitudinal at tibia, oriented in parallel to the muscle fascicles and perpendicular to the skin. The transducer’s alignment was considered satisfactory when several fascicles could be outlined across the image without interruption. The probe path was drawn on the skin with a pen according to the fascicle path seen from the real-time ultrasound image [22]. Panoramic images were obtained by moving the probe slowly along the marked line, as a continuous single view. Muscle fascicle length was measured as a linear trace from the lower aponeurosis to the upper aponeurosis (Motic Images Plus 2.0, Motic, Hong Kong, China). The lengths of three muscle fascicles crossing the shadow of the echo-absorptive marker at the middle and at the distal part of the GM, were averaged and subsequently used for analysis. Pennation angle was taken as the average angle of these three fascicles, measured at the point where they met the lower aponeurosis at the middle and the distal part of the GM. Muscle thickness was the perpendicular distance between the superficial and deep aponeuroses. Muscle thickness was measured at the middle and the distal part of the GM over two consecutive measurements and the average value was used for further analysis. (Figure 2). 

Following measurement of muscle architecture, resting ankle joint angle, was determined with the subjects at the same position. Reflective motion analysis markers were placed on the knee (femur-tibia joint line), ankle (lateral malleolus), and the fifth metatarsal to determine ankle joint angle at rest and during stretching. The position of the markers was recorded using a digital camera (Casio Exilim Pro EX-F1) placed perpendicular to the plane of motion of the leg, and ankle angle was calculated using free software (Tracker 4.91 © 2016 Douglas Brown, California, USA). Resting ankle angle was defined as the angle between the lines crossing the femur-tibia joint and lateral malleolus and the line defined by the down part of the heel and the end of the fifth metatarsal bone. The intra-class correlation coefficient (ICC) for resting ankle angle was 0.96 (95% confidence intervals (CI): 0.779–0.993, *p* = 0.000). 

Intersession reliability of ultrasound images was determined by comparing the analysis of the images obtained by 6 participants over two measurements on two separate days. The ICC for muscle fascicle length was 0.93 (95% CI: 0.670–0.988, *p* = 0.000), for muscular thickness it was 0.89 (95% CI: 0.500–0.981, *p* = 0.001), and for pennation angle, 0.81 (95% CI: 0.265–0.986, *p* = 0.013).

### 2.5. Muscle Architecture and Ankle Dorsiflexion Angle during Stretching 

Following the method described above, ultrasound images were obtained from the middle and the distal part of the GM muscle belly. Subsequently, athletes remained at a standing position for 2 min. After standing for 2 min, athletes performed a slow, passive standing gastrocnemius stretch (dorsiflexion), for 1 min, to the point of discomfort. A schematic representation of the study protocol is shown in Figure 1. Ultrasound panoramic views were obtained from the right GM during the 1 min passive standing gastrocnemius stretching intervention. Fascicle length, pennation angle and muscle thickness of GM of the right leg, were measured at the middle and the distal part of the GM muscle belly: 30% and 50% of the distance from the popliteal crease to the center of the medial malleolus respectively, marked on the skin [23]. Ultrasound probe was placed 38-mm above the skin marker identifying the middle part of the muscle belly. The probe path was drawn on the skin with a pen according to the fascicle path seen from the real-time ultrasound image [22]. The distance between the MTJ of the GM at rest and during stretching was defined as ‘‘MTJ displacement’’ [24]. Standing, ankle dorsiflexion stretching was performed with the participants barefoot, in a slow, continuous manner for 1 min. This stretch modality was chosen because it is commonly performed in sport environments [25]. Subjects were instructed to relax and to offer no active resistance to the movement. A 5 s pause was imposed before the end point of the static stretching, to allow still images to be acquired. To stretch the GM, the right foot was placed on the midline of a predetermined floor area with the heel at the edge of the area. The left foot was placed forward at a distance equal to the subject’s step length, which was measured in the familiarization session during gait. The subjects were asked to lean forward until the ankle attained dorsiflexion at the point of discomfort (80–90% of the maximum tolerate stretch) without heel lift or pelvic rotation. The hip and knee joints were maintained at an extended position during stretching. The subjects were asked to place their hands against the wall and to slowly bear their body weight on the foot to be tested [25]. Stretch intensity was determined based on the feedback from the subjects to ensure that stretch achieved the point of discomfort (rating 80 to 90, indicated by the athletes on a visual analogue scale of 0–100). Based on the same procedure used in prior investigations [26] the athletes were informed that 0 represented “no stretch discomfort at all” and 100 represented “maximal stretch discomfort”.

Following the method described above, maximal ankle dorsiflexion angle was measured using reflective motion analysis markers placed on the knee, ankle and fifth metatarsal. The angle between the horizontal and the line joining the knee and ankle markers was defined as the dorsiflexion angle. 

### 2.6. Statistical Analysis

Data are presented as means and standard deviations. The normality of data distribution was checked with the Shapiro–Wilks test. Between-groups differences, in anthropometric characteristics and architectural characteristics of GM at rest, were analyzed using unpaired T-test. A two-way analysis of variance (ANOVA) (time x group) with repeated measures for time (pre- and post-stretching) and group (rhythmic gymnasts or volley ball athletes) was conducted to examine the effect of stretching on all the examined variables (SPSS Statistics Version 22.0, IBM Corporation, Armonk, NY, USA). When a significant main effect or interaction was observed (*p* < 0.05) a Tukey’s post–hoc test was performed to examine significant differences between pairs of means. Effect sizes (ES) for the ANOVA were determined by partial eta squared (η^2^) (small: 0.01 to 0.059, moderate: 0.06 to 0.137, large > 0.138). For pairwise comparisons, ES was determined by Cohen’s *d* (trivial: 0–0.19, small: 0.20–0.49, moderate: 0.50–0.79 and large: 0.80 and greater) [27]. Test-retest reliability was assessed by calculating the intra-class correlation coefficients (ICCs). ICC values less <0.5, 0.5–0.75, 0.75–0.9, and >0.90 were considered indicative of poor, moderate, good, and excellent reliability, respectively. Statistical significance was set at *p* < 0.05.

## 3. Results

### 3.1. Resting Muscle Architecture and Ankle Joint Angle 

Rhythmic gymnasts displayed longer fascicles compared to the volleyball athletes, at mid-belly (t_18_ = 8.750, *p* = 0.000) and at the distal part of the GM (t_18_ = 4.646, *p* = 0.000) (by 20 and 18%, respectively) and greater resting ankle joint angle by 7% (t_18_ = 4.662, *p* = 0.000). The two groups had similar GM muscle thickness both at mid-belly (t_18_ = 1.411, *p* = 0.175) and distally (t_18_ = 1.248, *p* = 0.228) as well as distal pennation angle (t_18_ = −1.329, *p* = 0.201). Volleyball athletes demonstrated greater pennation angle compared to rhythmic gymnasts at mid-belly, by 10% (t_18_ = −3.185, *p* = 0.005). 

### 3.2. Muscle Architecture and Ankle Dorsiflexion Angle during Stretching

Rhythmic gymnasts displayed similar changes to volleyball athletes in pennation angles at mid-belly (24 vs. 28%, respectively) and at the distal part of GM (17 vs. 12%, respectively) (*p* > 0.145), and similar muscle thickness at mid-belly (9 vs. 5%, respectively) and at the distal part (51 vs. 64%, respectively) (*p* > 0.105) (Table 2). No difference was found between rhythmic gymnasts and volleyball athletes, in fascicle elongation at mid-belly (41 vs. 40%, respectively) (*p* = 0.063). However, gymnasts displayed greater fascicle elongation at the distal part of the GM (45 vs. 39%, respectively) (*p* = 0.026) greater maximal ankle dorsiflexion angle (51 vs. 43%, respectively) (*p* < 0.035) and MTJ displacement (*p* = 0.001) (Table 2). 

## 4. Discussion

The aim of this study was to examine whether elite rhythmic gymnasts display different architectural characteristics of GM at the middle and the distal part of the muscle belly and different ankle joint angles, at rest and during stretching, compared to volleyball athletes that do not engage in extensive flexibility training. The main findings of this study were that, compared with volleyball athletes, rhythmic gymnasts displayed greater resting fascicle length at the middle and at the distal part of the GM, greater fascicle elongation at the distal part of GM during stretching, greater MTJ displacement and greater ankle joint angles both, at rest and during dorsiflexion.

The greater fascicle length of the GM muscle in rhythmic gymnasts at mid-belly and at the distal part (by 20 and 18%, respectively) compared with volleyball players is an interesting finding of the present study. A number of cross-sectional studies indicated sport-specific profiles of fascicle length in athletes [28,29]. For example, elite track-and-field sprinters had greater vastus lateralis and GΜ fascicle lengths compared to control subjects [28], and the same was found in sprinters’ fascicle length of the lateral gastrocnemius compared to controls [29]. Although cross-sectional studies cannot exclude the possibility of inherited profiles in athletes, long-term, systematic sport-specific training may have a substantial impact on muscle architecture. Recently, Moltubakk et al. [30] compared ballet dancers to controls. The authors found longer GM fascicles in resting prone position in ballet dancers and suggested that long-term flexibility training may induce adaptations at the sarcomere level [30]. 

However, stretching interventions reported equivocal results. In a recent meta-analysis, Freitas et al. [31] reported that short-term stretching interventions (3–8 weeks) in adults do not change muscle and tendon architectural properties although they increase muscle extensibility and tolerance to a greater tensile force. In contrast, Simpson et al. [10] found increased medial and lateral gastrocnemii fascicle length and greater pennation angles following 6-weeks of loaded static stretching training. Nevertheless, an increase in fascicle length following stretching has not yet been clearly demonstrated in humans and longitudinal studies are needed to examine long-term adaptations to stretching training and distinguish them from factors such as genetic predisposition and sport selection [30].

Fascicle elongation was measured at mid-belly and at the distal part of GM during maximal ankle dorsiflexion. Previous studies found greater elongation in flexible compared to inflexible subjects [30,32] and stretching interventions studies also reported an increased muscle extensibility after 3 or 4 weeks of static stretching training [11,24]. In the present study, rhythmic gymnasts demonstrated similar fascicle elongation to volleyball players at mid-belly (41 vs. 40%, respectively) and, although the effect size was large (*d* = 0.94), the difference did not reach statistical significance (*p* = 0.063, Table 2). A possible explanation of this result may be participants training history. Although volleyball players were not submitted to extensive flexibility training, they were high-level athletes who also performed other types of training, such as plyometric exercises, and eccentric training that may have increased muscle extensibility, while in previous studies, flexible subjects were compared to active controls [30].

Interestingly, significantly greater elongation was observed in rhythmic gymnasts at the distal part of the GM (45 vs. 39%, respectively) (*p* = 0.026, *d* = 1.15, Table 2) and significantly greater MTJ displacement. It is reported that architectural adaptations may be specific to certain regions of the muscle [33]. Longitudinal variations in fibre length [34] and orientation have been shown to occur along a given muscle [33]. The commonly used location for longitudinal measures of the GM is at 30% of the straight-line distance from the popliteal crease to the centre of the medial malleolus [21]. However, fascicle length at mid-belly may not reflect muscle architecture accurately across the entire muscle [17] therefore in the present study, the 50% of the same distance (distal) was also assessed. Zhang et al. [17] reported that passive fascicle lengths were longer distally compared to proximally during ankle dorsiflexion. Also, a recent stretching intervention in adults found that lateral and medial gastrocnemii muscle fascicles in the musculotendinous junction lengthened more than those in the muscle belly (by 25 and 5% respectively) after 6 weeks of stretching training [10]. De Monte et al. [35] examined GM fascicle elongation during ankle dorsiflexion and reported that the displacement of the point located on the MTJ was significantly greater than that of the point located on the aponeurosis. Collectively, it is possible that architectural adaptations of the GM may occur non-homogeneously along the length of the muscle. In animal studies it has been shown that sarcomere number is adjusted so as to achieve an optimum sarcomere length where a muscle experiences its highest levels of tension [36] and Jakobsen et al. [37] reported that a great proportion of the muscle fibres near the MTJ seem to undergo remodelling.

In the present study, rhythmic gymnasts had greater ankle angle at rest, in prone position by 7%, compared to volleyball players (Table 2). Resting angle joint angle is influenced by the sum of all the torques acting around the joint [30]. In a recent study comparing triceps surae architectural properties in ballet dancers and controls, Moltubakk et al. [30] found similar resting ankle joint angle between the two groups and the authors assumed that the different operating length of the dancers, depended on tissue material properties and did not imply different slack length of the tissues surrounding the joint. Blazevich et al. [32] also reported no differences in the ankle joint angle anatomical position between flexible and inflexible subjects, however, the authors did not examine the flexibility training background of the participants. Τhe mechanisms underpinning the different resting ankle joint angle between rhythmic gymnasts and volleyball athletes cannot be explained by the present study design. Albeit speculative, the greater resting ankle angle may imply a different slack length, due to long-term, extensive flexibility training. However, further research is needed to examine if long-term systematic flexibility training and/or other training regimes may alter the ‘neutral’, resting ankle joint angle. Moltubakk et al. [30] pointed out that ‘neutral’ ankle joint angle may not be a valid starting point for all populations for measuring elongation or the different components of the muscle tendon unit. 

Maximal ankle dorsiflexion range of motion was significantly greater in rhythmic gymnasts compared to the volleyball athletes (51 vs. 43%, respectively) (Table 2). Maximal ankle dorsiflexion angle was defined by the end point of the motion that all athletes could achieve. Greater joint ROM has also been reported in previous studies in flexible vs. inflexible subjects [30,32]. Moltubakk et al. [30] also reported greater ankle dorsiflexion ROM in ballet dancers compared to controls, a fact consistent with the higher volume of stretching performed during their training practices. The longer GM fascicles and a greater maximal fascicle elongation during stretch may facilitate the greater maximal ankle dorsiflexion angle observed in rhythmic gymnasts [30]. 

## 5. Conclusions

In conclusion, greater ankle angles at rest and during dorsiflexion were observed in elite rhythmic gymnasts compared to volleyball players, longer GM fascicles at the middle and at the distal part of the GM and greater muscle elongation at the distal part of GM during static stretching. These findings highlight that muscle architectural properties differ between athletes with different flexibility and that there may be non-uniform adaptations along GM length depending on training history. However, this study design limits interpretation of these findings. Chronic flexibility training may be part of the stimulus inducing muscle architectural adaptations however, other factors, such as heredity may also influence muscle architecture.

## Figures and Tables

**Table 1:** Anthropometric characteristics of the participants (means ± standard deviation).

Anthropometric Characteristics	Rhythmic Gymnasts (*n* = 10)	Volleyball Players (*n* = 10)	*t* (18)	*p*
Age (y)	21.3 ± 1.6	24.3 ± 4.7	−1.918	0.071
Competitive experience (y)	12.2 ± 1.2	10.8 ± 1.9	1.976	0.064
Height (cm)	169.9 ± 2.9	173.6 ± 4.3	−2.264	0.036
Body mass (Kg)	56.7 ± 8.4	70.6 ± 14.7	−2.595	0.018
Calf length (cm)	40.8 ± 1.4	42.5 ± 2.3	−1.962	0.065
Leg length (cm)	86.8 ± 2.5	88.1 ± 1.7	−1.427	0.171
Weekly training (h/week)	32.0 ± 4.2	9.9 ± 1.4	15.763	0.000

*p*: statistical significance, *t*: t-statistic.

**Table 2:** Changes in muscle architecture characteristics and ankle range of motion (ROM) following stretching for the rhythmic gymnasts (*n* = 10) and the volleyball athletes (*n* = 10).

Parameter	Group	Pre-Stretching Measurements	Stretching Measurements	*p*	Cohens’ *d* (pre vs. Stretching)	Δ Values (pre vs. Stretching)	Cohens’ *d* of Δ Values between Groups
**Fascicle Length** **Mid-belly (cm)**	Rhythmic	5.93 ± 0.27	8.37 ± 0.68	0.063	4.97	2.44 ± 0.59	0.94
	Volleyball	4.74 ± 0.33†	6.62 ± 0.81†		3.20	1.88 ± 0.67	
**Fascicle Length** **Distal part (cm)**	Rhythmic	5.63 ± 0.52	8.14 ± 0.74	0.026	4.14	2.51 ± 0.54	1.15
	Volleyball	4.57 ± 0.51†	6.31 ± 0.91†		2.49	1.74 ± 0.84	
**Thickness** **Mid-belly (cm)**	Rhythmic	2.36 ± 0.66	2.56 ± 0.62	0.105	0.33	0.20 ± 0.15	0.79
	Volleyball	2.04 ± 0.21	2.16 ± 0.22		0.59	0.11 ± 0.08	
**Thickness** **Distal part (cm)**	Rhythmic	1.04 ± 0.27	1.51 ± 0.24	0.868	1.94	0.47 ± 0.16	0.06
	Volleyball	0.86 ± 0.36	1.34 ± 0.44		1.26	0.48 ± 0.18	
**Pennation angle** **Mid-belly (⁰)**	Rhythmic	22.4 ± 2.5	16.9 ± 1.7	0.145	2.66	5.4 ± 2.2	0.72
	Volleyball	25.8 ± 2.4†	18.7 ± 3.1		2.71	7.2 ± 2.9	
**Pennation angle** **Distal part (⁰)**	Rhythmic	19.0 ± 2.5	15.8 ± 2.4	0.478	1.42	3.2 ± 2.0	0.32
	Volleyball	20.5 ± 2.6	18.0 ± 3.7		0.81	2.5 ± 2.8	
**Ankle ROM** **(⁰)**	Rhythmic	121.7 ± 4.1	59.1 ± 4.6	0.001	15.24	62.6 ± 2.7	4.60
	Volleyball	113.2 ± 3.7†	65.0 ± 4.7†		12.00	48.2 ± 3.8	
**MTJ Displacement (cm)**	Rhythmic					3.15 ± 0.56	1.43
	Volleyball					2.31 ± 0.67 †	

†: *p* < 0.001 from the corresponding value in Rhythmic gymnasts. Ankle ROM: from resting ankle joint angle (pre-stretching measurements) to maximal ankle dorsiflexion angle (stretching measurements).
